# Demographic and educational factors associated with COPD knowledge among primary family caregivers: A cross-sectional study at a tertiary hospital

**DOI:** 10.1097/MD.0000000000044228

**Published:** 2025-09-05

**Authors:** Nu Wang, Yu Zhu, Ye Zhang

**Affiliations:** a Department of Respiratory and Critical Care Medicine, Hejiang People’s Hospital, Luzhou City, Sichuan Province, China.

**Keywords:** caregiver knowledge, COPD, educational intervention, health literacy, logistic regression

## Abstract

Chronic obstructive pulmonary disease (COPD) requires effective management that often depends on the knowledge level of primary family caregivers. This study assessed caregiver knowledge using a validated scale and identified key factors associated with higher COPD knowledge. A cross-sectional survey was conducted from April 2020 to January 2024 at a tertiary hospital in Southwest China. A total of 726 primary family caregivers of recently discharged COPD patients completed the general information questionnaire and the COPD health knowledge scale. Participants were categorized into high and low knowledge groups based on a predefined cutoff score of 21, reflecting adequate disease-related knowledge. Multivariate logistic regression was used to identify independent predictors. Higher educational attainment (OR = 126.568, 95% CI: 37.489–427.302, *P* < .001), receipt of COPD-specific health education (OR = 114.731, 95% CI: 33.426–393.804, *P* < .001), and older age (OR = 1.058 per year increase, 95% CI: 1.025–1.093, *P* < .001) were significantly associated with higher COPD knowledge. The predictive model showed excellent discrimination (C-index = 0.995) and good calibration, although external validation is needed to confirm its generalizability. This study identified education level, health education exposure, and age as key factors influencing caregiver COPD knowledge. The use of a fixed cutoff score enhances interpretability and external comparability. Targeted educational interventions may improve caregiver knowledge and support better disease management. Further longitudinal and interventional studies are warranted to confirm causal relationships.

## 1. Introduction

Chronic obstructive pulmonary disease (COPD) is a leading cause of morbidity and mortality worldwide, posing significant challenges to healthcare systems due to its progressive nature and the complex management it requires.^[1–3]^ Effective management of COPD not only relies on the clinical interventions provided by healthcare professionals but also significantly on the role of family caregivers.^[4–6]^ These caregivers often assume responsibilities that include medication administration, symptom monitoring, and lifestyle adjustments critical to managing COPD.^[7–9]^ Thus, the knowledge and preparedness of caregivers play a crucial role in the quality of life and clinical outcomes of COPD patients.

The knowledge level of caregivers is influenced by various factors, including demographic characteristics, educational background, and access to health education. Previous studies have highlighted that caregivers with higher educational attainment and those who have received specific health education tend to be better equipped to manage COPD.^[10–12]^ However, there remains a gap in understanding the comprehensive factors that contribute to caregivers’ knowledge levels, especially in diverse healthcare settings.

This study aims to characterize the demographic and educational variations associated with the knowledge levels of primary family caregivers of COPD patients. By identifying these associations, we seek to inform future research directions and the design of targeted educational strategies. By focusing on caregivers of recently discharged patients from a tertiary hospital, this research seeks to capture a critical period where effective disease management and application of knowledge are paramount. Through a cross-sectional survey design, this study provides valuable insights into the demographic and educational determinants of COPD knowledge among caregivers, aiming to inform targeted interventions that can enhance caregiver education and support, ultimately improving patient outcomes.

The findings from this study will contribute to the growing body of literature on caregiver roles in chronic disease management and highlight the importance of tailored educational programs. By identifying the key predictors of COPD knowledge, healthcare providers can develop more effective strategies to support caregivers, ensuring that they are well-prepared to meet the demands of managing COPD and enhancing the overall care provided to patients.

## 2. Methods

### 2.1. Study design and setting

This cross-sectional survey was conducted at a tertiary hospital from April 2020 to January 2024. The study targeted primary family caregivers of COPD patients who had been recently discharged. This approach was chosen because it allowed access to caregivers who were actively involved in the day-to-day management of COPD post-discharge, reflecting a critical period for effective disease management and knowledge application.

Participant recruitment and data collection caregivers were identified from hospital records and contacted via telephone by trained nurses who explained the study objectives. Those who agreed to participate were either mailed the survey package or visited by the survey team, depending on their preference and accessibility. This method ensured that the data collected reflected the caregivers’ current knowledge and practices, crucial for assessing the immediate applicability of their COPD management skills.

### 2.2. Survey instruments

#### 2.2.1. General information questionnaire

The general information questionnaire was designed to capture key demographic and caregiving-related characteristics of the participants. It collected data on age, gender, relationship to the patient (spouse, child, other relative, non-relative), marital status (married, divorced, widowed, single), educational level (no formal education, primary education, secondary education/high school, tertiary education), monthly income (<3000 RMB, 3000–5000 RMB, >5000 RMB), medical payment method (fully self-paid, partially reimbursed, fully reimbursed), duration of caregiving (<1 year, 1–5 years, >5 years), smoking status (smoker, nonsmoker), and whether the caregiver had received specific COPD health education (yes/no).

#### 2.2.2. COPD health knowledge scale

The COPD health knowledge scale used in this study was adapted from the Bristol COPD knowledge questionnaire and consists of 40 items covering 7 domains: COPD disease knowledge, causes of COPD, symptoms of COPD, knowledge about lung infections and exacerbations, pulmonary rehabilitation, smoking-related knowledge, and treatment knowledge (including antibiotics, inhaled bronchodilators, home oxygen therapy, and corticosteroids). Participants answered each item with “yes,” “no,” or “don’t know.” Each correct response was awarded 1 point, while incorrect or “don’t know” responses received 0 points, resulting in a total possible score ranging from 0 to 40, with higher scores indicating greater knowledge about COPD. Example items include “COPD is a preventable and treatable disease,” “regular exercise worsens COPD,” and “antibiotics are effective for viral infections.” The reliability and validity of the scale were rigorously evaluated prior to its application, with internal consistency confirmed by a Cronbach alpha of 0.88, test-retest reliability showing a coefficient of 0.93, and content validity supported by a content validity index of 0.84.

### 2.3. Ethical considerations

This study was approved by the Institutional review Board of Hejiang County People’s Hospital (ethics approval number: 2024-001). All procedures were conducted in accordance with the ethical standards of the responsible committee and with the 1964 declaration of Helsinki and its later amendments. Written informed consent was obtained from all participants prior to data collection. Participation was voluntary, and all data were anonymized to ensure confidentiality.

### 2.4. Statistical analysis

Participants were categorized into low and high COPD knowledge groups based on a pre-defined cutoff of 21 points on the COPD health knowledge scale, where scores ≥ 21 were considered indicative of adequate knowledge. This threshold was selected based on the scale’s design characteristics and prior studies suggesting that scores above 70% of the total reflect satisfactory disease understanding. This approach avoids sample-specific bias and improves the generalizability of our findings. Those with scores below the median were categorized as having low COPD knowledge, while those with scores at or above the median were categorized as having high COPD knowledge. For group comparisons between the low and high COPD knowledge groups, the Mann–Whitney *U* test was applied to continuous variables (e.g., age) due to non-normal distributions, while Chi-square or Fisher exact tests were used for categorical variables (e.g., marital status, educational level, smoking status). To construct the multivariate logistic regression model, we adopted a backward elimination approach guided by the Akaike information criterion (AIC) rather than relying solely on univariate *P*-values. While initial univariate analyses were conducted to explore potential associations, all clinically relevant variables were included as candidates in the full model regardless of their univariate significance. Variables were then sequentially removed based on their AIC contribution, allowing the model to retain predictors that may exert joint effects only observable in multivariable contexts. This method improves model parsimony, reduces overfitting, and enhances generalizability, particularly in observational studies where collinearity or confounding may obscure individual associations. This approach aligns with current best practices in clinical prediction modeling as outlined by Chowdhury and Turin (2020).^[13]^ All statistical analyses were performed using SPSS version 26.0 (IBM Corp., Armonk), and 2-sided *P*-values <.05 were considered statistically significant.

## 3. Results

### 3.1. Characteristics of participants by COPD knowledge level (based on cutoff score)

Table 1 summarizes the characteristics of study participants categorized into low and high COPD knowledge levels based on a predefined cutoff score of 21 points on the COPD health knowledge scale, with scores ≥21 reflecting adequate disease understanding. A significant difference in age was observed between the 2 groups, with the high knowledge group exhibiting a higher median age of 53.42 years (IQR: 43.94–62.56) compared to 44.37 years (IQR: 34.63–55.15) in the low knowledge group (*P* < .001). No significant differences were found in the caregiver’s relationship to the patient (*P* = .228), with children being the most common caregiver type across both groups. Marital status differed substantially, as only 4.5% of participants in the low knowledge group were married compared to 47.5% in the high knowledge group (*P* < .001). Similarly, educational attainment showed significant disparity: 47.1% of participants in the high knowledge group had education at the junior college level or below, while 42.8% of those in the low knowledge group had high school education or above (*P* < .001). Monthly income and medical payment methods did not differ significantly between the groups (*P* = .188 and *P* = .101, respectively). Years of caregiving experience were similarly distributed, with no statistically significant difference (*P* = .980). In contrast, a marked difference was observed in the receipt of COPD-specific health education, with 48.3% of participants in the High knowledge group having received such education compared to only 5.6% in the low knowledge group (*P* < .001), highlighting the potential role of targeted education in enhancing disease-related knowledge among caregivers.

**Table 1:** Characteristics of participants by COPD knowledge level (cutoff score = 21).

Characteristics	Low knowledge (<21)	High knowledge (≥21)	*P* value
*n*	342	384	
Age, median (IQR)	44.37 (34.63, 55.15)	53.42 (43.94, 62.56)	<.001
Relationship with patient, *n* (%)	0.228		
Spouse	42 (5.8%)	49 (6.7%)	
Child	251 (34.6%)	296 (40.8%)	
Other	49 (6.7%)	39 (5.4%)	
Marital status, *n* (%)	<0.001		
Married	33 (4.5%)	345 (47.5%)	
Divorced/widowed/other	309 (42.6%)	39 (5.4%)	
Educational level, *n* (%)	<0.001		
High school and above	311 (42.8%)	42 (5.8%)	
Junior college and below	31 (4.3%)	342 (47.1%)	
Monthly income, *n* (%)	0.188		
<3000 RMB	41 (5.6%)	31 (4.3%)	
3000–5000 RMB	259 (35.7%)	299 (41.2%)	
>5000 RMB	42 (5.8%)	54 (7.4%)	
Medical payment method, *n* (%)	0.101		
Fully self-paid	28 (3.9%)	39 (5.4%)	
Partially reimbursed	273 (37.6%)	316 (43.5%)	
Fully reimbursed	41 (5.6%)	29 (4.0%)	
Years of care, *n* (%)	0.980		
<5 yr	31 (4.3%)	36 (5.0%)	
5–10 yr	276 (38.0%)	310 (42.7%)	
>10 yr	35 (4.8%)	38 (5.2%)	
Received COPD-specific health education, *n* (%)	<0.001		
No	301 (41.5%)	33 (4.5%)	
Yes	41 (5.6%)	351 (48.3%)	

Participants were categorized into low and high COPD knowledge groups based on a predefined cutoff score of 21 on the COPD health knowledge scale, consistent with prior literature identifying this threshold as indicative of sufficient disease-related knowledge.

% = percentage of the total group, COPD = chronic obstructive pulmonary disease, IQR = interquartile range, *n* = number of participants.

### 3.2. Univariate and multivariate analysis of participant characteristics and their association with COPD knowledge levels

The final multivariate logistic regression model retained 5 predictors following backward elimination guided by AIC, which included age, marital status, educational level, and receipt of COPD-specific health education. Table 2 presents the results of univariate and multivariate logistic regression analyses exploring the relationship between various characteristics and COPD knowledge levels among 726 participants. Age significantly predicts higher COPD knowledge, with an odds ratio (OR) of 1.037 (95% CI: 1.026–1.048) in the univariate analysis and 1.058 (95% CI: 1.025–1.093) in the multivariate analysis, both *P* < .001. The relationship with the patient shows no significant association with knowledge levels; spouses serve as the reference group, with children and others showing ORs of 1.011 (95% CI: 0.648–1.578) and 0.682 (95% CI: 0.379–1.229), respectively. Marital status reveals that being divorced, widowed, or otherwise not married is significantly associated with lower knowledge levels compared to being married, with ORs of 0.012 (95% CI: 0.007–0.020) in the univariate and 0.013 (95% CI: 0.004–0.040) in the multivariate analysis, both *P* < .001. Educational level is also a strong predictor, with individuals having a junior college or lower education showing dramatically higher ORs of 81.691 (95% CI: 50.104–133.191) univariate and 126.568 (95% CI: 37.489–427.302) multivariate, both *P* < .001, compared to those with a high school education or above. Monthly income and medical payment methods show no statistically significant impacts on COPD knowledge levels in the adjusted model. Finally, having received specific health education on COPD is associated with a substantially higher likelihood of greater knowledge, with ORs of 78.086 (95% CI: 48.150–126.636) in the univariate and 114.731 (95% CI: 33.426–393.804) in the multivariate analysis, both *P* < .001.

**Table 2:** Univariate and multivariate analysis of participant characteristics and their association with COPD knowledge levels.

Characteristics	Total (N)	Univariate analysis		Multivariate analysis	
		Odds ratio (95% CI)	*P* value	Odds ratio (95% CI)	*P* value
Age	726	1.037 (1.026–1.048)	**<.001**	1.058 (1.025–1.093)	**<.001**
Relationship with patient	726				
Spouse	91	Reference			
Child	547	1.011 (0.648–1.578)	.962		
Other	88	0.682 (0.379–1.229)	.203		
Marital status	726				
Married	378	Reference		Reference	
Divorced/widowed/other	348	0.012 (0.007–0.020)	**<.001**	0.013 (0.004–0.040)	**<.001**
Educational level	726				
High school and above	353	Reference		Reference	
Junior college and below	373	81.691 (50.104–133.191)	**<.001**	126.568 (37.489–427.302)	**<.001**
Monthly income	726				
<3000 RMB	72	Reference		Reference	
3000–5000 RMB	558	1.527 (0.930–2.505)	.094	1.581 (0.390–6.405)	.521
>5000 RMB	96	1.700 (0.918–3.150)	.092	5.254 (0.903–30.566)	.065
Medical payment method	726				
Fully self-paid	67	Reference		Reference	
Partially reimbursed	589	0.831 (0.498–1.386)	.478	2.616 (0.631–10.846)	.185
Fully reimbursed	70	0.508 (0.257–1.002)	.051	3.378 (0.429–26.567)	.247
Years of care	726				
<5 yr	67	Reference			
5–10 yr	586	0.967 (0.583–1.606)	.897		
>10 yr	73	0.935 (0.481–1.817)	.843		
Received COPD specific health education	726				
No	334	Reference		Reference	
Yes	392	78.086 (48.150–126.636)	**<.001**	114.731 (33.426–393.804)	**<.001**

N: total number of participants. Odds ratio (95% CI): odds ratio with 95% confidence interval, indicating the likelihood of higher COPD knowledge associated with each characteristic compared to the reference group. *P* value: statistical significance of the odds ratio. Reference: baseline category used for comparison in the odds ratio calculations. Bold values indicate statistical significance (*P* < .05). Univariate analysis: analysis considering each variable independently. Multivariate analysis: analysis considering all variables simultaneously to adjust for potential confounders.

COPD = chronic obstructive pulmonary disease.

### 3.3. Statistical evaluation of the predictive model for COPD knowledge

**Figure 1.**
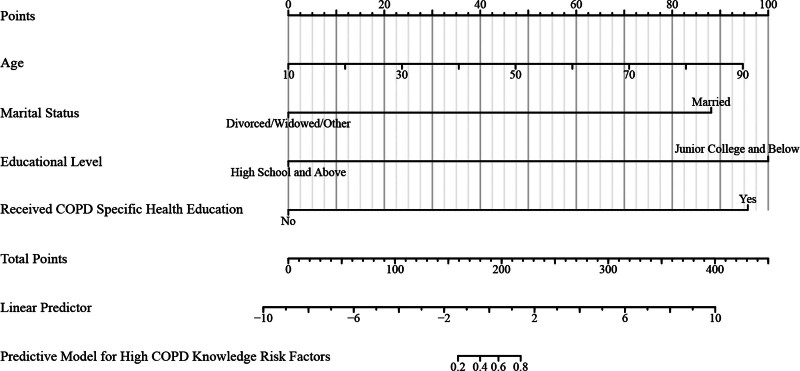
Predictive model for high COPD knowledge risk factors. This figure represents a diagnostic line chart that visually delineates the contribution of various factors to predicting high COPD knowledge. Each factor (age, marital status, educational level, and receipt of specific health education) is plotted against its weight in points, illustrating its relative influence on the overall risk prediction. The “total points” line aggregates the contributions from all factors, which correlates directly with the “linear predictor” to assess risk. COPD = chronic obstructive pulmonary disease.

**Figure 2.**
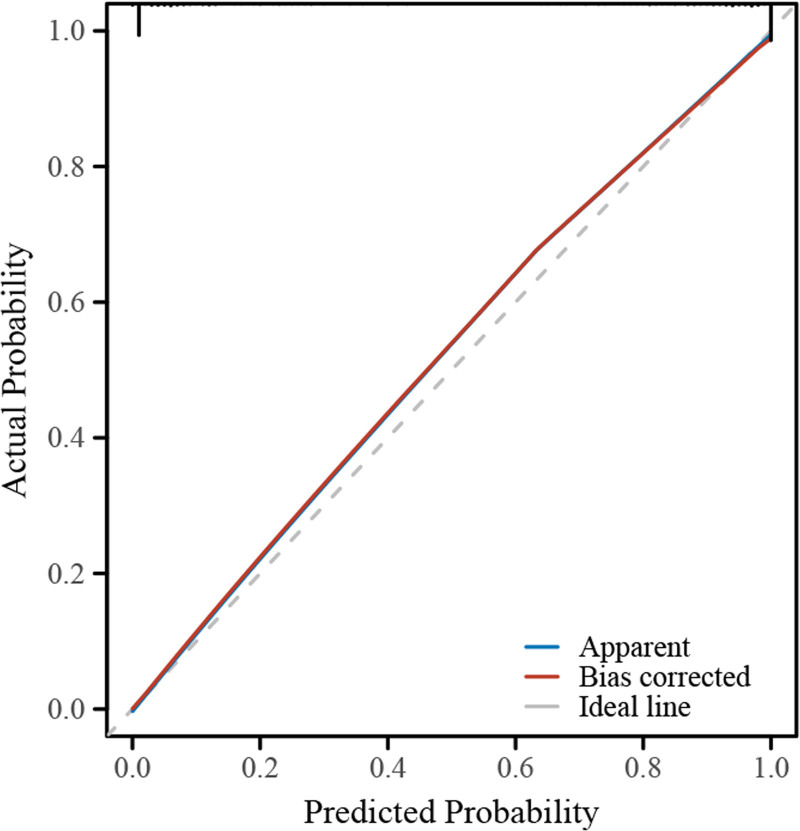
Diagnostic calibration curve for COPD knowledge prediction model. This figure displays the calibration curve for the predictive model, comparing the actual probabilities of high COPD knowledge against the predicted probabilities. The “apparent” line shows the model’s calibration based on the data used for modeling. The “bias corrected” line adjusts for any overfitting within the model, providing a more realistic prediction accuracy. The “ideal line” represents a perfect prediction where the predicted probabilities exactly match the actual probabilities, serving as a benchmark for assessing model performance. COPD = chronic obstructive pulmonary disease.

Table 3 provides a comprehensive statistical evaluation of the predictive model for COPD knowledge, elaborating on the model’s testing, discrimination, and calibration. The likelihood ratio test, presented in the model testing segment, shows a Chi-square value of 874.91 (*P* < .001), indicating a highly significant fit of the model to the data. This robust model fit supports the factors selected for prediction, as visualized in Figure 1, where different predictors like age, marital status, educational level, and receipt of specific health education are quantified for their impact on predicting high COPD knowledge. The model’s discrimination ability, measured by the concordance index (C-index), is exceptionally high at 0.995 (95% CI: 0.991–0.998, *P* < .001). This indicates an excellent capability of the model to distinguish between individuals with low and high COPD knowledge levels, as demonstrated in Figure 1. The predictive accuracy is reflected in the linear predictor scores which correlate highly with the actual knowledge levels, showing that the model effectively separates the high knowledge group from the low knowledge group. Further, the calibration of the model, assessed by the Goodness-of-fit test, results in a Chi-square value of 3.9067 with a *P*-value of .8654, suggesting that there is no significant departure from a perfect fit. This indicates that the predicted probabilities of COPD knowledge are well-aligned with the observed probabilities. Figure 2 illustrates this alignment with its calibration curve, comparing the apparent, bias-corrected, and ideal lines. The close proximity of the model’s calibration curve to the ideal line indicates that the model predictions are unbiased and closely approximate the true probabilities, further validating the model’s utility in clinical or educational settings where accurate prediction of COPD knowledge is critical. These combined results from Table 3, along with the visual evidence from Figures 1 and 2, underscore the strength and reliability of the predictive model in assessing the risk factors associated with high COPD knowledge. The model demonstrates high statistical performance within the sample; however, due to the lack of external validation, its practical applicability remains to be determined. Future studies should aim to validate this model in independent caregiver populations to confirm its robustness and utility.

**Table 3:** Statistical evaluation of the predictive model for COPD knowledge.

Evaluation aspect	Evaluation metric	Statistic	*P*-value
Model testing	Likelihood ratio test	Chi-square: 874.91	<.001
Discrimination	Concordance index (C-index)	C-index: 0.995 (0.991–0.998)	<.001
Calibration	Goodness-of-fit test	Chi-square: 3.9067	.8654

Model testing assesses the overall fit of the model using the likelihood ratio test. Discrimination measures the model’s ability to distinguish between different outcome classes using the concordance index (C-index). Calibration evaluates the agreement between observed outcomes and predictions using the goodness-of-fit test. Chi-square: a statistical test that measures the discrepancy between observed and expected outcomes. C-index: a measure of the predictive accuracy of the model, ranging from 0.5 (no better than chance) to 1.0 (perfect separation). *P*-value indicates the statistical significance of the test results, with values close to 0 suggesting strong evidence against the null hypothesis.

COPD = chronic obstructive pulmonary disease.

## 4. Discussion

The findings of this study provide a descriptive analysis of demographic and educational factors associated with variations in COPD knowledge among primary family caregivers. These results offer valuable insights that can guide the development of future educational strategies. Our results align with existing literature and provide further insights into the complex dynamics influencing caregiver knowledge and the implications for COPD management.

Our study revealed that higher educational levels and receipt of specific COPD health education are strongly associated with higher knowledge levels among caregivers. This is consistent with the findings of Matarese et al (2021), who conducted a thematic synthesis of qualitative studies and highlighted that caregivers contribute significantly to the self-care of COPD patients through education and knowledge acquisition.^[14]^ Their study emphasized that caregivers play a crucial role in maintaining disease stability and promoting healthy behaviors, particularly during the stable phases of COPD. This supports our observation that caregiver exposure to targeted educational content is positively associated with knowledge levels. While promising, the direct efficacy of such interventions requires confirmation through prospective studies. Bagnasco et al (2021) explored the experiences and support needs of informal caregivers during acute exacerbations of COPD.^[15]^ They found that caregivers often feel unsupported by healthcare professionals and experience high levels of stress and anxiety. Our study’s findings, which show a significant association between higher knowledge levels and receipt of specific health education, suggest that providing comprehensive education and support to caregivers can alleviate some of these stresses and improve their ability to manage exacerbations effectively. This highlights the need for healthcare professionals to engage more proactively with caregivers, offering both technical and psychological support to enhance their caregiving capacity. Marques et al (2021) conducted a systematic literature review and identified that interventions to support informal caregivers of COPD patients are generally narrow in scope and not specifically designed to address caregivers’ unique needs.^[16]^ Our study contributes to this body of knowledge by demonstrating the positive impact of tailored educational interventions on caregivers’ knowledge levels. The strong internal performance of our predictive model, as indicated by the C-index of 0.995, suggests potential for further development. However, we acknowledge the need for validation in independent datasets before any conclusions can be drawn regarding its clinical or educational use. The integrative review by Zhang et al (2024) highlights the heavy psychological and physical burden faced by caregivers of COPD patients and the role of resilience and coping strategies in managing this burden.^[17]^ Our findings, which emphasize the importance of education, suggest that enhancing caregivers’ knowledge can be a critical component of their coping strategy, potentially reducing their burden and improving their resilience. By equipping caregivers with the necessary knowledge and skills, healthcare systems can better support these individuals, reducing the overall strain on both caregivers and patients.

In response to increasing recognition of the limitations of *P*-value–based variable selection, our modeling strategy incorporated backward elimination guided by the AIC. This approach allowed us to consider the joint predictive value of multiple variables, reducing the risk of excluding clinically relevant but statistically non-significant variables. As emphasized by Chowdhury and Turin (2020),^[13]^ variable selection should balance statistical performance with clinical interpretability, and reliance on univariate *P*-values alone may lead to biased estimates and unstable models. By applying AIC-based selection, we sought to enhance the robustness and replicability of our findings, particularly in the context of developing predictive models for caregiver knowledge in COPD.

The insights from our study have significant implications for clinical practice. Healthcare providers may consider developing and implementing educational programs tailored to the needs of COPD caregivers, although further research is needed to evaluate their effectiveness. This includes not only disease-specific knowledge but also practical skills for managing symptoms and preventing exacerbations. As Pendoni et al^[11]^ highlighted, caregivers’ contributions are essential for maintaining disease stability and ensuring a normal life for patients. Therefore, comprehensive caregiver education programs should be integrated into COPD management plans to enhance both patient and caregiver outcomes. Furthermore, Bahadori et al (2023) demonstrated that smartphone-based pulmonary rehabilitation education can significantly decrease caregiver burden and improve their quality of life.^[18]^ Our study supports the expansion of such innovative educational interventions, leveraging technology to deliver accessible and effective education to caregivers. This approach can be particularly beneficial in reaching a broader caregiver population, providing continuous support, and fostering better disease management practices.

In the original analysis, COPD knowledge levels were stratified based on the sample median. While statistically straightforward, such a data-dependent threshold may reduce external validity. Therefore, in the revised analysis, we applied a fixed cutoff score (≥21 points) on the COPD health knowledge scale, which corresponds to at least 70% of the maximum score and indicates a sufficient level of disease-specific understanding. This decision enhances the generalizability of our results and aligns with common interpretive practices for health knowledge scales in caregiver populations.

This study provides valuable evidence on the determinants of disease-related knowledge among primary family caregivers of patients with COPD; however, several methodological and contextual limitations must be acknowledged. First, the cross-sectional study design precludes any inference of causality between potential predictors – such a s age, education level, or exposure to COPD-specific health education – and knowledge outcomes. While associations were statistically significant, the temporal sequence between exposure and outcome remains unclear. Future longitudinal or interventional studies are needed to confirm causal relationships and assess the impact of educational interventions on knowledge acquisition and retention. Second, although we adopted a backward elimination approach guided by the AIC – a widely recommended technique for balancing model parsimony and predictive performance – residual confounding remains a possibility. This is particularly relevant in observational studies where unmeasured or imprecisely measured factors (e.g., health literacy, cognitive function, access to information) may influence both the independent variables and the outcome. Third, the dichotomization of COPD knowledge scores using a fixed cutoff value of 21, based on prior validation studies of the COPD health Knowledge scale, improves external comparability and interpretability. However, it also entails a loss of information and potential misclassification, especially for participants whose scores cluster around the threshold. Given that health knowledge is inherently continuous and influenced by context-specific norms, future studies should consider incorporating sensitivity analyses with alternate thresholds, latent class models, or modeling knowledge as a continuous outcome to capture more nuanced differences. Fourth, the generalizability of our findings is limited by the single-center sampling frame. All participants were recruited from a tertiary teaching hospital in Southwest China, which may not reflect the demographic, educational, and socioeconomic diversity of caregivers in rural settings, community hospitals, or other regions. Broader sampling strategies are needed to ensure the model’s applicability across settings with varying healthcare infrastructures. Finally, the use of self-reported measures to assess COPD knowledge may be subject to recall bias or social desirability bias. Although the COPD health knowledge scale has been previously validated in Chinese populations, it may not fully capture practical disease management knowledge or skills. Future research should consider incorporating objective assessments or performance-based tasks to complement subjective evaluations and provide a more comprehensive understanding of caregiver competence.

In conclusion, our study highlights the significant associations between caregiver education, receipt of health education, and COPD knowledge levels, providing a foundation for future interventional studies. By identifying demographic and educational factors associated with caregiver knowledge, we provide a foundation for future research aimed at designing and testing targeted interventions to improve disease management and patient outcomes. The integration of comprehensive educational programs into COPD care plans is essential to support caregivers, reduce their burden, and ultimately enhance the quality of care provided to COPD patients.

## Author contributions

**Conceptualization:** Nu Wang, Yu Zhu, Ye Zhang.

**Data curation:** Nu Wang, Yu Zhu, Ye Zhang.

**Formal analysis:** Nu Wang, Yu Zhu, Ye Zhang.

**Investigation:** Ye Zhang.

**Methodology:** Yu Zhu, Ye Zhang.

**Project administration:** Ye Zhang.

**Software:** Yu Zhu, Ye Zhang.

**Supervision:** Ye Zhang.

**Validation:** Yu Zhu, Ye Zhang.

**Visualization:** Yu Zhu, Ye Zhang.

**Writing – original draft:** Nu Wang, Yu Zhu, Ye Zhang.

**Writing – review & editing:** Yu Zhu, Ye Zhang.
